# Beyond the Pericapsular Nerve Group (PENG) Block: A Narrative Review

**DOI:** 10.5152/TJAR.2021.21230

**Published:** 2022-06-01

**Authors:** Ana Sofia Teles, Ece Yamak Altınpulluk, Rajendra Kumar Sahoo, Felice Galluccio, Diego García Simón, İlker İnce, Marilina Olea, Emilio González-Arnay, Carlos Salazar, Karla Espinoza, Mario Fajardo-Pérez

**Affiliations:** 1 Morphological Madrid Research Center (MoMaRC), Madrid, Spain; 2 Department of Anaesthesiology, Instituto Português de Oncologia Do Porto Francisco Gentil, Porto, Portugal; 3 Department of Outcomes Research Anaesthesiology Institute Cleveland Clinic, Cleveland, OH, USA; 4 Anaesthesiology Clinical Research Office, Atatürk University, Erzurum, Turkey; 5 Kalinga Institute of Medical Sciences, Bhubaneswar, India; 6 Division of Rheumatology, Department of Medical-Geriatric, University Hospital AOU Careggi, Florence, Italy; 7 Department of Anaesthesiology, Hospital Universitario de Móstoles, Móstoles, Spain; 8 Department of Anaesthesiology and Reanimation, Atatürk University Faculty of Medicine, Erzurum, Turkey; 9 Hospital Interzonal General Dr. José Penna, Bahía Blanca, Buenos Aires, Argentina; 10 Department of Anatomy, Histology and Neuroscience, Autonomous University of Madrid, Madrid, Spain; 11 Department of Basic Medical Sciences, University of La Laguna, Tenerife, Canary Islands, Spain; 12 Hospital Universitario 12 de Octubre, Madrid, Spain; 13 Department of Anaesthesiology, Hospital Mexico Costa Rica, San Jose, Costa Rica

**Keywords:** nerve block, pain management

## Abstract

The pericapsular nerve group block shows promising results in providing pain relief with a potential motor-sparing effect in hip fracture patients. In this narrative review, we analyze the published articles, and we describe the structures achieved when performing the block. We conducted a literature search to identify the articles performing the pericapsular nerve group block, in the adult or paediatric population, from November 1, 2018, to May 15, 2021. Of the 68 selected articles, 38 were considered eligible, including 1 double-blinded randomized comparative trial, 4 observational studies, and 33 case series and case reports. The technique was described in both acute and chronic pain settings, mainly performed as single shot. All studies described effective analgesia. Quadriceps weakness was experienced in some patients. It has been described as easy to perform and has a low rate of complications. It lacks, however, adequately powered randomized controlled trials to assess its clinical value and efficacy.

## Introduction

Over the last few years, regional anaesthesia has witnessed the emergence of various novelties in ultrasound-guided techniques. The pericapsular nerve group (PENG) block, firstly described in 2018 by Girón-Arango et al^1^ promptly caught the attention with its promising results in providing pain relief in the perioperative setting and a potential motor-sparing effect in patients with hip fractures. It targets the most richly innervated segment of the hip joint, the anterior capsule.

Advantages of peripheral nerve blocks over systemic analgesia for pain treatment after hip fractures were described recently in a Cochrane review.^2^ Compared to systemic analgesia, the authors reported on pain on movement reduction of 3.4 in a 10-points Numerical Rating Scale 30 minutes after block placement, reduced time to first mobilization, reduced complications secondary to immobilization, like pneumonia, and higher patient satisfaction. 

The use of peripheral nerve blocks for pain management after a hip fracture has a long and diverse history, including the fascia iliaca, femoral nerve (FN), psoas compartment, obturator nerve (ON), or lateral femoral cutaneous nerve (LFCN) blocks. 

Since its description, several reports or letters have been published hypothesizing the mechanism of action of the PENG block, efficacy, local anaesthetics (LA) distribution and optimal doses, and the inadvertent risk of motor weakness. Additionally, its applicability now travels beyond the hip pathology and new settings have been described.

The literature available on the PENG block is still scarce and, in this review, we analyze the published articles related to the technique in different clinical scenarios, the LA used, its effectiveness, and complications reported, and we further describe the structures achieved when performing the PENG block. 

## Relevant Anatomy

In 1997, Birnbaum et al^3^ reported on the FN’s articular branches to innervate the anterolateral hip joint capsule and the articular branches of the ON to be responsible for the anteromedial portion. The posterior and inferior portions were innervated by branches from the sciatic or superior gluteal nerves. Gerhardt et al^4^ in a histological study, established a predominant presence of nociceptive fibers in the anterior and superolateral portions of the hip joint capsule. In the posterior and inferior sections, the neural fibers found were identified as mechanoreceptors. 

An accessory obturator nerve (AON), present in 10%-30% of patients, has also been reported to consistently innervate the hip joint capsule.^5^ It arises either from the ventral branches of the L3 and L4 spinal nerves or the ON directly and travels by the psoas major at its medial margin, crosses the superior pubic ramus dorsal to the pectineus muscle, over the iliopubic eminence (IPE), to terminate on the hip capsule.

Thus, the 3 nerves (FN, ON, and AON) are assumed to be the primary mediators of pain in patients with hip fractures, suggesting that these should be the main targets for hip analgesia. This premise was recently confirmed by Short et al^6^ in their anatomical study. The authors describe superior accountability from the AON and FN than formerly postulated in the innervation of the anterior portion of the capsule. Furthermore, this study identified the relevant landmarks for those articular branches. Between the anterior inferior iliac spines (AIIS) and the IPE, one could consistently find the high articular branches of the FN and the AON, whereas closer to the inferomedial acetabulum, the articular branches of the ON. This was the core information that was endorsed in the development of the PENG block. It aims at blocking those branches while taking advantage of ultrasound technology.

## Technique Description

The procedure is performed under ultrasound guidance using a curvilinear low-frequency ultrasound probe in the supine position. After identifying the AIIS with the probe positioned in a transverse plane, the probe should be rotated approximately 45° to be in the orientation of the pubic ramus. When reaching this position, the authors describe the identification of the iliopsoas muscle and tendon sitting on the IPE, the femoral artery, and the pectineus muscle, more medially.^1^

## Methods

In this narrative review, we performed a literature search using PubMed, MEDLINE, Cochrane Library, and Google Scholar for published articles related to the PENG block, from November 1, 2018, to May 15, 2021, in the English language. We used the keywords “pericapsular nerve group,” “peri-capsular nerve group,” and “PENG block.” We included clinical trials, observational studies, case series, case reports, and letters to the editor, describing the procedure either in the adult or pediatric population. Excluded articles included those other than the English language, duplicate articles, and articles not related to the PENG block. Initially, 68 articles were identified. Of these, 6 articles were excluded based on their titles or abstracts if they were not associated with the PENG block. A comprehensive reading of the 62 articles was completed, of which 24 were excluded for not being relevant in the context of the present review or not a technique description in adult or paediatric patients.

**Figure 1.**
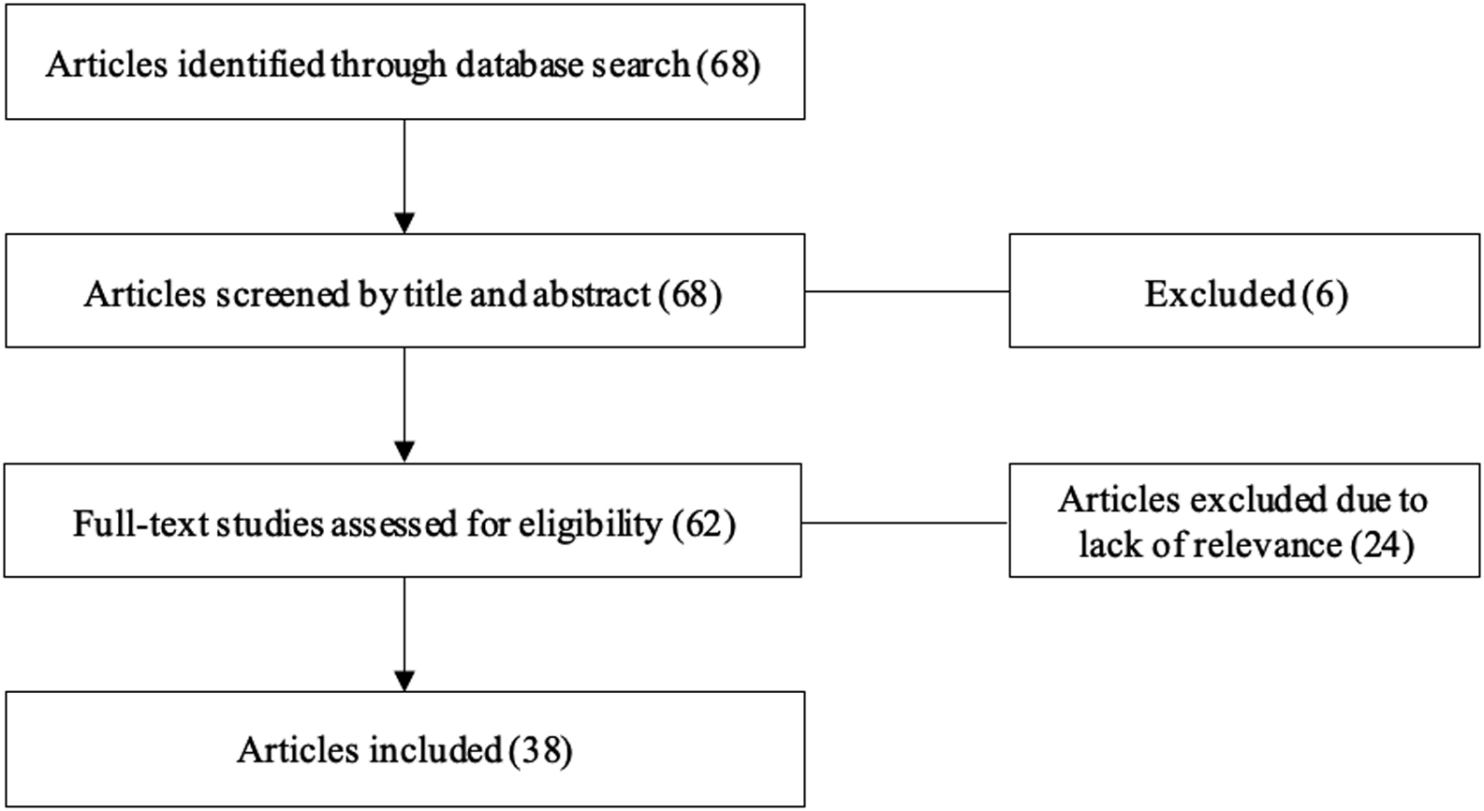
Algorithm of the selection of articles in different phases for this review.

At last, 38 studies were included (Figure 1), and data were extracted related to the number of individuals involved, the intervention performed, the type of LA used, catheter introduction or single-shot, associated regional anaesthesia techniques, application in acute or chronic pain setting, pain scores, complications, and other relevant topics.

## Results

The studies this review comprises include 1 double-blinded randomized comparative trial,^7^ 4 observational studies,^8–11^ 13 cases series,^1,12–23^ and 20 case reports.^24–43^

Lin et al^7^ compared the PENG block with the FN block for hip surgery analgesia. The authors randomly allocated 60 patients with hip fracture presenting for surgery to receive the PENG block or the FN block (20 mL of 0.75% ropivacaine were used in both). In the post-anaesthesia care unit (PACU), 90% of the patients in the PENG group experienced none or mild pain, compared to 57% in the FN block group (*P*  = .04). In the latter group, 33% of the patients showed reduced quadriceps strength on day 1, compared to 7% in the PENG group (*P*  = .004). Finally, 97% of the PENG group patients were satisfied with the analgesia received, in comparison to 70% in the FN block group (*P * = .02).

In a prospective study, Sahoo et al^11^ aimed at evaluating the decrease in pain score at rest and with passive movement after PENG block, and the efficacy of the analgesia when sitting hip fracture patients for spinal anaesthesia. Thirty minutes after the administration of 20 mL of 0.25% bupivacaine with 4 mg dexamethasone, the mean pain score decreased from 7.45 ± 1.53 (mean ± SD) to 1.1 ± 1.07 at rest and from 9.45 ± 0.75 to 2.35 ± 1.34 with passive movement (*P* < .001). Optimal positioning was achieved in 75% of patients (Supplementary Table 1).

Supplementary tables 2 to 4 summarize the results of all case reports and case series, totaling 122 patients. The block was performed in the acute pain setting in 30 reports (118 patients) and it was mainly performed in the context of hip surgery. Indications also included 2 cases of vein ligation and stripping, a case of transurethral resection of laterally located bladder tumors, and a hip and thigh vaso-occlusive crisis, due to a sickle cell disease. The single-shot technique was the most regularly used, including 25 reports and 94 patients (Supplementary Table 2). Local anaesthetics used included lidocaine, ropivacaine, bupivacaine, and levobupivacaine, in different concentrations, with 4 mg of dexamethasone in some cases. The volume administered ranged from 10 to 40 mL. Sensory loss of the FN, ON, LFCN, and genitofemoral nerve was frequently reported after performing the block.^13,27,29,33,36,43^ No complications were reported, apart from 3 cases of quadriceps motor weakness and inability to perform a straight leg raise.^29,33^ Local anaesthetics used in these scenarios were 15 mL 0.5% bupivacaine plus 15 mL 2% lidocaine, 20 mL 0.5% bupivacaine with 1 : 400 000 epinephrine and 50 µg mL^−1^ dexamethasone and 20 mL 0.25% bupivacaine with 1 : 400 000 epinephrine and 50 µg mL^−1^ dexamethasone.

Five reports (24 patients) used continuous LA infusion after insertion of a catheter, also in acute pain setting (Supplementary Table 3). The catheter was kept for 1-3 days, with perfusion of 5-6 mL h^−1^. Pain scores were inferior to 3 out of 10 points. Three cases of intravenous catheter placement were reported.

Three reports (4 patients) describe the PENG block application for chronic pain treatment (Supplementary Table 4), with decreased pain scores and no complications.

Paediatric use of the PENG block was evaluated in 4 case reports for acute pain management, with good outcomes, including one that used a continuous infusion through a catheter.^34,37,38,42^

## Discussion

The literature available on the PENG block, while mainly considering perioperative analgesia for hip surgery, has been showing that the procedure offers effective pain relief with preserved quadriceps strength.

Lin et al^7^ tested the PENG block in a double-blinded randomized comparative fashion, conceding it as a possible ideal regional technique for hip surgery. Such technique should allow a significant pain reduction while avoiding delayed mobilization and discharge. The authors found the technique to be more successful than FN block in controlling pain and preventing quadriceps weakness. 

Aydin et al^28^ described a new indication for the PENG block, beyond the hip pathology. The patients went through vein ligation and stripping in the segment of both FN and ON dermatomes under an effective PENG block for surgical anaesthesia. The authors used 30 mL of LA and reported to have effectively blocked not only the FN and ON dermatomes but also the LFCN and genitofemoral nerve dermatomes, in both patients. They proposed that, when using higher volumes than the 20 mL originally described, the PENG block will resemble a lumbar plexus block.

The skin of the lateral thigh can be anaesthetized after blocking the LFCN. This procedure is vastly performed and is of pertinence for hip surgery. The nerve enters the thigh just below the inguinal ligament and is typically found 10-15 mm medial to the anterior superior iliac spine (ASIS). It then passes laterally over the sartorius muscle, where it divides into multiple branches to supply the skin of the thigh on its lateral aspect as far distal as the knee.^44^ The block can be performed after ultrasound-guided infiltration immediately under the inguinal ligament 1-2 cm medial to the ASIS, with a high success rate.^45^ When preparing to perform the PENG block, with the probe aligned parallel to the inguinal ligament, the anatomical structures to execute the LFCN block can be easily identified by displacing the probe in an oblique direction toward lateral and superior until the ASIS is seen. The nerve should appear between the tensor fasciae latae muscle and sartorius muscle or superficial to the latter.

Additionally, Santos et al^46^ reported a successful case of a PENG block with a perineural catheter, with the tip being placed between the psoas tendon anteriorly and the pubic ramus posteriorly. The authors described, as well, that analgesia beyond the FN and ON territories was observed, namely in the LFCN. Similarly, Yamak Altinpulluk et al^47^ reported on the possibility to reach the LFCN when performing a PENG block, after injecting different volumes of LA in the targeted area. This LFCN coverage seems to be an additional potential of the PENG block, supported by the previously published articles.

Based on a recent anatomic study,^6^ the PENG block was developed to target the articular branches of the FN and AON between the AIIS and IPE.^1^ The authors reported having accomplished their purpose, although being unable to assess the LA medial spreading to reach the interfascial plane between the pectineus and obturator externus muscles. This subpectineal plane corresponds to the location of the articular branches of ON and is perceptible using ultrasound. It was previously described as a target point for the ON.^48^ According to that previously described technique, Nielsen et al^49^ conducted a cadaveric study to evaluate the proximal spread around the ON articular hip branches after ultrasound-guided injection in the subpectineal plane. The authors demonstrated that injection of 15 mL of dye spread proximally, with a considerably high success rate, into the obturator canal and to the ON and AON articular branches to the hip capsule. The dye consistently spread dorsally through the pectineus muscle to reach its lateral limit, where the AON typically is located and, when present, it spread around the nerve.

**Figure 2.**
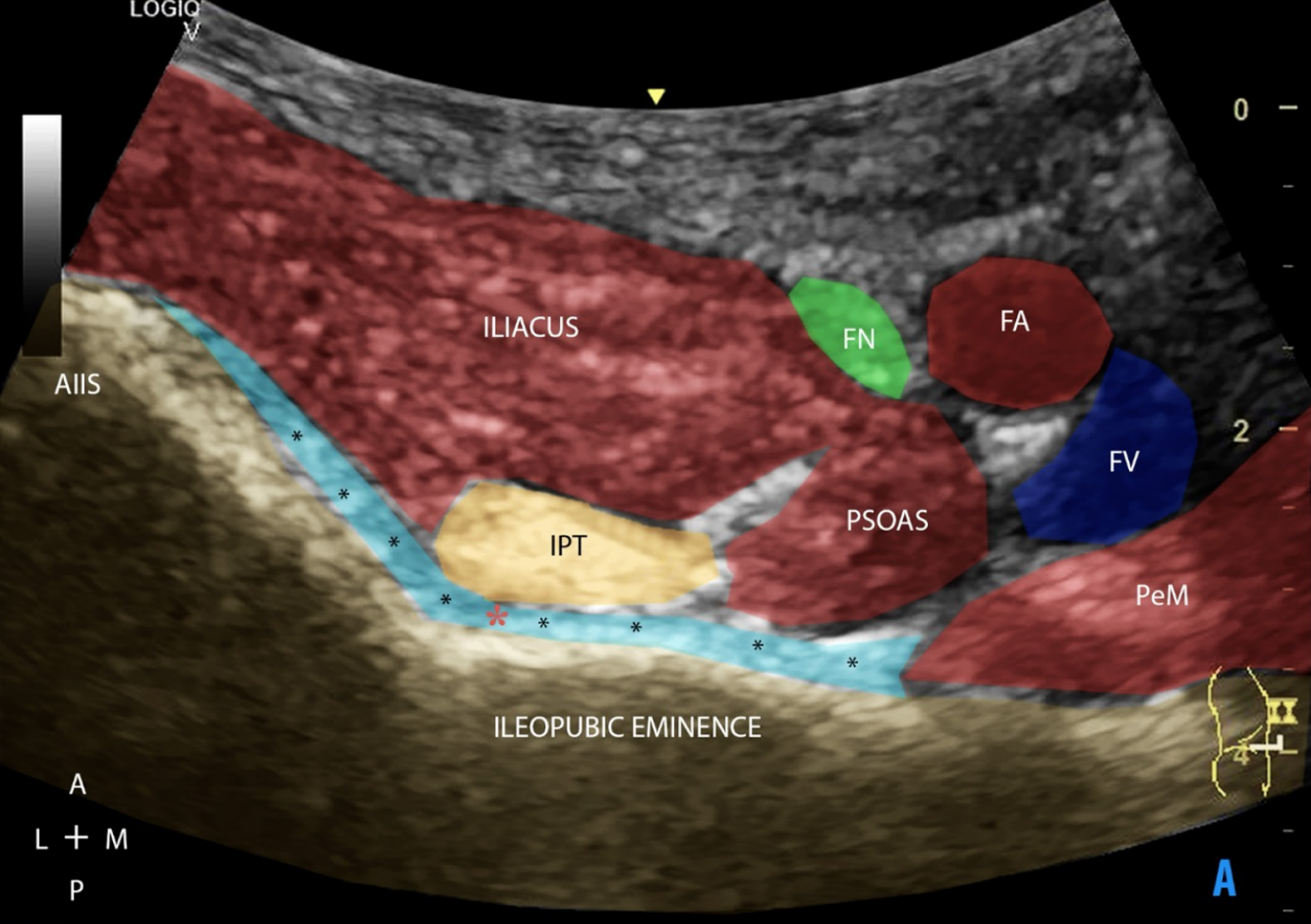
Ultrasound illustration of the region of pertinence to the PENG block. AIIS, anterior inferior iliac spine; FN, femoral nerve; FA, femoral artery; FV, femoral vein; SM, sartorius muscle; IPT, iliopsoas tendon; PeM, pectineus muscle.

One could assume that, given the spreading pattern after this ultrasound-guided subpectineal technique, which shows consistent dispersion along the deep face of the pectineus muscle to reach its lateral margin, the reverse trajectory may be well accomplished when executing the PENG block. Figure 2 shows the structures present in the targeted area.

Although the analgesic effect of the PENG block was notable and reproducible in different centers,^18,28,33^ the distribution of injectate relative to the hip capsule articular branches innervating the joint was not defined. This question was addressed^50^ and 10 and 20 mL of dye were injected in cadavers, to conclude that the anterior portion of the hip capsule, identified as the nociceptive segment, was stained after the spread of the dye through the bursal space between the iliopsoas and anterior hip joint capsule. The authors concluded that this pattern supports the “true” pericapsular nature of the PENG block, by reaching the articular branches of the FN, ON, and AON. Another cadaveric study using 10 mL of contrast showed the contrast to the extent to the joint space and its accumulation in the lower joint recess.^47^

## Conclusion

The main issue with the PENG block is the lack of adequately powered RCT comparing it with the more commonly used epidural or other peripheral nerve blocks.

The PENG block is increasingly recruiting supporters with its, although limited to case series, encouraging results and motor-sparing effect, denoting a significant benefit. It may arise as an easier and safer good alternative to other techniques, such as the lumbar plexus block. The latter exemplifies an advanced procedure more difficult to accomplish, with a higher rate of complications associated.

Because the PENG block is a recently described technique, further investigations assessing its clinical value, efficacy, and the diffusion of the solution to confirm the coverage area need to be developed, as well as to determine its optimal volume and additional indications.

## Figures and Tables

**Supplementary Table 2.** Reported Cases of PENG Block Single Shot for Acute Pain Management

	**N**	**Procedure**	**LA**	**Additional RA interventions and LA**	**Results**	**Complications**
Girón-Arango et al	5	Analgesia for hip fracture	B 0.25% 20 mL w/ 1:400 000 epinephrine or R 0.5% 20 mL w/ 1:200 000 epinephrine and 4 mg dexamethasone	None	All patients could flex the hip and perform a straight leg raise to 15º and reported significantly reduced pain scores at 30 minutes. No quadriceps weakness	Not reported
Luftig et al	3	Analgesia for hip fracture	B 0.5% w/ epinephrine 20 mL + normal saline 10 mL	None	Effective analgesia within 30 minutes. Restored ability to range the hip with minimal/no pain	Not reported
Acharya et al	10	Analgesia for hip fracture	B 0.125% 20 mL w/4 mg dexamethasone	None	Lower pain score: 1-3/10 at 10 minutes 9 patients were able to self-position for sitting SAB	Not reported
Roy et al	10	Hip surgery	LA not specified	LFCN block (LA not specified)	In the group without LFCN block, “a few patients” required rescue opioids for dermatomal pain Authors describe a combination of the PENG block with LFCN block to provide better analgesia than the PENG block alone	Not reported
Kukreja et al	12	THA	R 0.5% 20 mL	None	Postoperative pain scores and OME lower in the group undergoing primary THA compared to revisions	Not reported
Ince et al	3	THA	B 0.5% 10 mL + L 2% 10 mL	LESP block: B 0.25% 30 mL	No pain in the incision site and in the sensory areas of the FN, LFCN and ON at 1 hour. Pain score <3/10 for 24 hours6-8 mg IV morphine equivalent consumption	Not reported
Fusco et al	4	THA	LB 0.375% 20 mL w/4 mg dexamethasone	LIA: LB 0.375% 20 mL, 30 mg ketorolac and 0.1 mg epinephrine	Postoperative pain at rest was “2 controls” and with movement was “4 controls” during 24 hours No additional analgesics needed	Not reported
Sandri et al	10	THA	LB 0.25% 40 mL w/4 mg dexamethasone	LIA: LB 0.25% 80 mL, 30 mg ketorolac, 0.1 mg epinephrine, 10 mg morphine Surgical incision: M 1% 10 mL	Postoperative pain score <4/10No postoperative opioids required	Not reported
Mistry et al	5	Analgesia for hip fracture	Not specified	None	All patients reported significant dynamic pain relief in 15 minutes No quadriceps weakness	Not reported
Orozco *et al*	5	Hip arthroscopy	B 0.75% 10 mL + L 1% 10 mL	FNB: B 0.75% 10 mL + L 1% 10 mL	Pain score 0-24 h <3/10 and 48-72h <1/10. No opioids required.	Not reported
Casas Reza et al	8	Hip surgery	LB 0.375% 20 mL	LFCN block: LB 0.375% 5 mL	No motor block of the FN or ON Need for IV morphine in first 24 hours (3-6 mg) in 2 cases	Not reported
Ahiskalioglu et al	2	TURBT	B 0.5% 15 mL + L 2% 15 mL	None	No adductor muscle spasm. Loss of sensation of ON, FN, LFCN and genitofemoral nerve	Not reported
Aydin et al	2	Vein ligation and stripping	B 0.5% 15 mL + L 2% 15 mL	None	Surgery tolerated with a maximum of 2 mg/kg/h propofol No opioids required	Not reported
Aksu et al	1*	Open reduction congenital hip dysplasia	B 0.25% 10 mL	None	Need for single-dose ibuprofen 10 hour postoperatively No additional analgesia required for 24 hours	Not reported
Wyatt et al	1*	Hip and thigh vaso-occlusive crisis (SCD)	B 0.25% 16 mL w/ dexmedetomidine	FNB: B 0.25% 8 mL w/ dexmedetomidine	Lower pain score: 0/10. No opioids required over 24 hours and the patient was able to ambulate After block dissipation, the pain score 0-2/10 with oral medication (11 mg IV morphine equivalent) Discharged at 48 hour	Not reported
Fusco et al	1	THA	LB 0.375% 15mL	None	No additional analgesics required during the perioperative period	Not reported
Ahiskalioglu et al	2	Vein ligation and stripping Hip surgery	B 0.5% 15mL + L 2% 15 mL	None	Sensory loss of LFCN, genitofemoral, anterior femoral cutaneous, ON, and saphenous nerves No need for additional analgesics Lower pain score: 1/10 at 10 minutes and 2/10 with positioning (hip fracture case)	Quadriceps weakness and inability to achieve a straight leg raise (1 case)
Thallaj et al	1	THA	B 0.25% 30 mL	LFCN block: B 0.25% 5 mL	Pain score 0/10 during 24 hour, 2/10 at rest and 3/10 with movement at 24-48 hours No additional analgesics	Not reported
Fusco et al	1	Analgesia for hip fracture	LB 0.375% 20 mL w/ 4 mg dexamethasone	None	Lower pain score: 1/10 with passive mobilization and 4/10 at rest in the lying position at 24 hours No need for analgesic for 12 hours	Not reported
Bilal et al	2	Hip surgery	B 0.25% 30 mL	None	Postoperative pain score <3/10 during 24 hoursNo rescue analgesia	Not reported
Yu et al	2	THA	B 0.25% or 0.5% 20 mL w/ 50 mcg/mL dexamethasone and 1:400,000 epinephrine	None	Postoperative opioids required Decreased sensation over the distal half of the thigh, knee and saphenous nerve distribution below the knee	Quadriceps weakness and inability to perform a straight leg raise
Orozco et al	1*	Femoral osteosynthetic material removal	B 0.5% 10 mL w/ epinephrine	FNB: L 1% 7.5 mL + LB 0.75% 7.5 mL LFCN block: L 1% 2.5 mL 1% L + LB 0.75% 2.5 mL	Pain score <2/10 for 72 hoursNo additional analgesia required	Not reported
Ahiskalioglu et al	1	Medial thigh mass removal	B 0.5% 15 mL + L 2% 15 mL	None	Sensory loss of the FN, ON, and LFCN dermatomes at 5 minutes No intraoperative additional opioids and sedatives required besides 1 mg midazolam Discharge on the same day	Not reported
Talawar et al	1	Hip arthroscopy	B 0.5% 10 mL + L 2% 10 mL w/ epinephrine	LFCN block: B 0.5% 5 mL + L 2% 5 mL w/ epinephrine	Sensory loss of the anterior, medial, and lateral thigh at 20 minutes Analgesia lasted for 4:30 hours postoperatively	Not reported
Oksüz et al	1	Distal tibia and fibula fracture surgery	B 0.25% 25 ml + L 2% 10 ml	Subgluteal block: B 0.25% 15 ml	Adequate anaesthesia of FN, ON, LFCN, and SN upon evaluation at 30 minutes 2 h surgery and no surgical or tourniquet pain	Not reported

PENG, pericapsular nerve group; LA, local anaesthetic; RA, regional anaesthesia; B, bupivacaine; R, ropivacaine; SAB, subarachnoid block; LFCN, lateral femoral cutaneous nerve; THA, total hip arthroplasty; OME, oral morphine equivalent; L, lidocaine; LESP, lumbar erector spine plane; FN, femoral nerve; ON, obturator nerve; IV, intravenous; LB, levobupivacaine; LIA, local infiltration analgesia; M, mepivacaine; FNB, femoral nerve block; TURBT, transurethral resection of lateral located bladder tumors; SCS, sickle cell disease; SNB, sciatic nerve block; SN, sciatic nerve.

*Paediatric patient.

**Supplementary Table 3.** Reported Cases of Continuous PENG Block for Acute Pain Management

	**N**	**Procedure**	**LA**	**Catheter Infusion**	**Results**	**Complications**
Del Buono et a*l*	10	Hip surgery	R 0.375% or L 0.5% 20 mL	Not specified Stopped at 72 hours	Lower pain score: 2/10 at 20 minutes. 2/10 median pain score after 12, 24, and 48 hours No rescue medication needed	3 cases of IV catheter placement
Singh et al	10	Hip surgery	B 0.25% 20 mL	B 0.25% at 5 mL h-1 at the end of the surgery Stopped on day 2	Lower pain score: 1-3/10 at 30 minutes, <2/10 at 6 h and <1/10 at 48 hNo additional analgesics required intraoperative or postoperatively	Not reported
Singh et al	1	THA	B 0.5% 15 mL	B 0.5% at 5 mL h-1 at the beginning of the surgery, continued at the end of the surgery w/ B 0.125% at 5 mL h-1 Stopped on day 3	Sensory loss of the FN, ON, LFCN, and genitofemoral nerve at 30 minutes Pain score always <3/10, not requiring additional analgesia	Not reported
Wyatt et al	1*	Analgesia for hip fracture	B 0.25% 14 mL	R 0.1% at 6 mL h-1 at the end of the surgery Stopped at 24 hours	Pain score of 2-3/10 after surgery, pre-infusion No motor weakness FLACC 0 at 12 hours	Not reported
Fujino et al	2	THA	R 0.5% 20 mL	R 0.2% at 6 mL h-1 before the end of the surgery Stopped at 48 hours	Pain score of 0/10 at rest and ≤3/10 at movement during first 48 hoursBromage scale 0	Not reported

PENG, pericapsular nerve group; LA, local anaesthetic; R, ropivacaine; L, lidocaine; IV, intravenous; B, bupivacaine; THA, total hip arthroplasty; FN, femoral nerve; ON, obturator nerve; LFCN lateral femoral cutaneous nerve; FLACC, face, legs, activity, cry, consolability.

*Paediatric patient.

**Supplementary Table 4.** Reported Cases of PENG Block for Chronic Pain Management

	**N**	**Procedure**	**Neurolytic PENG Block**	**Results**	**Complications**
Jaramillo et al	2	Refractory pain (hip osteoarthritis)	Bipolar RF ablation 60 seconds at 80° once at 2-3 different locations using a 4-output RF generator	Pain score decreased from 10/10 and 7/10 to 0–1/10 at 3 months No need for opioid analgesia and subjective improvement in the quality of life Repeated in 9 months in 1 patient due to pain recurrence.	Not reported
Romero et al	1	Analgesia for hip fracture (metastatic carcinoma)	6% phenol 10 mL	Pain score pre-block of 8/10. Complete pain relief at 30 minutes, maintained at 2 weeks No motor block	Not reported
Pimenta et al	1	Analgesia for hip metastasis of gastric cancer	5% phenol 4 mL	Severe refractory pain pre-block (10/10) 90% pain relief until patient’s death (10 days later)	Not reported

PENG. pericapsular nerve group; RF, radiofrequency.

**Supplementary Table 1. t1-tjar-50-3-167:** Reported Studies of the PENG Block

	**Study Type**	**N**	**Procedure**	**Groups**	**LA**	**Primary Outcome**	**Results**	**Complications**
Lin et al	Double-blinded randomized comparative trial	66	Hip surgery	PENG vs FNB	PENG: R 0.75% 20 mL FNB: R 0.75% 20 mL	Pain score	PENG group: less pain in the PACU, better preserved quadriceps strength	Reduced/absent quadriceps strength in 33% in the PENG group vs 77% in FNB group
Sahoo et al	POS	20	Analgesia for hip fracture	PENG	B 0.25% 20 mL w/ 4 mg dexamethasone	Pain control and ease of sitting for SAB	Pain score at rest <3/10; in 80%, mild pain with movementOptimal sitting in 75%	Not reported
Remily et al	ROS	96	THA	SAB + FIB + PENG (immediately postprocedure) vs SAB + FIB	PENG: B 0.5% 10 mL w/ 1:400 000 epinephrine FIB: B 0.5% 30 mL w/ 1:200 000 epinephrine	Patient outcome, pain score and opioid consumption	Shorter LOS, farther initial distance walked, lower pain scores until the 48-hour mark, longer time to first opioid, less opioid on day 1, 2 and cumulatively over the entire stay	Not reported
Kukreja et al	ROS	16	Revision THA	PENG + QLB vs QLB	PENG: R 0.5% 20 mL QLB: B 0.25% 25 mL	Pain score and opioid consumption	PENG + QLB group: lower pain at 6 h and 24 h and lower opioid use until 12 h	Not reported
Mysore et al	ROS	123	THA	PENG + LIA vs LIA	PENG: B 0.25% 20 mL w/ 1:200 000 epinephrine and 2 mg dexamethasone LIA: B 0.25% 20-40 mL w/ epinephrine	Opioid consumption	PENG block + LIA group: reduction in the mean 24 h postoperative opioid consumption	Not reported

PENG, pericapsular nerve group; LA, local anaesthetic; RCT, randomized controlled trial; FNB, femoral nerve block; PACU, post-anaesthesia care unit; R, ropivacaine; ROS, retrospective observational study; THA, total hip arthroplasty; LIA, local infiltration analgesia; B, bupivacaine; POS, prospective observational study; SAB, subarachnoid block; FIB, fascia iliaca block; LOS, length of stay; QLB, quadratus lumborum block.
